# Optimal Access Point Power Management for Green IEEE 802.11 Networks †

**DOI:** 10.3390/s21062076

**Published:** 2021-03-16

**Authors:** Rosario G. Garroppo, Gianfranco Nencioni, Luca Tavanti, Bernard Gendron, Maria Grazia Scutellà

**Affiliations:** 1 Department of Ingegneria dell’Informazione, Università di Pisa, 56122 Pisa, Italy; rosario.garroppo@unipi.it; 2 Department of Electrical Engineering and Computer Science, University of Stavanger, 4021 Stavanger, Norway; 3 CUBIT, Consortium Ubiquitous Technologies, 56021 Cascina, Italy; luca.tavanti@cubitlab.com; 4 CIRRELT and DIRO, Université de Montréal, Montréal, QC H3T 1N8, Canada; bernard.gendron@cirrelt.ca; 5 Department of Informatica, Università di Pisa, 56127 Pisa, Italy; scut@di.unipi.it

**Keywords:** wireless LAN, energy efficiency, resource allocation, optimisation, mixed integer non-linear programming, green networking, network management

## Abstract

In this paper, we present an approach and an algorithm aimed at minimising the energy consumption of enterprise Wireless Local Area Networks (WLANs) during periods of low user activity. We act on two network management aspects: powering off some Access Points (APs), and choosing the level of transmission power of each AP. An efficient technique to allocate the user terminals to the various APs is the key to achieving this goal. The approach has been formulated as an integer programming problem with nonlinear constraints, which comes from a general but accurate characterisation of the WLAN. This general problem formulation has two implications: the formulation is widely applicable, but the nonlinearity makes it NP-hard. To solve this problem to optimality, we devised an exact algorithm based on a customised version of Benders’ decomposition method. The computational results proved the ability to obtain remarkable power savings. In addition, the good performance of our algorithm in terms of solving times paves the way for its future deployment in real WLANs.

## 1. Introduction

The energy saving issue in wireless networks is currently the focus of many research activities. For example, there is a plethora of works dealing with analysing and reducing the power consumption in cellular networks [1,2], in wireless sensor networks [3,4], in wireless mesh networks [5,6], and in Wireless Local Area Networks (WLANs). With specific focus on IEEE 802.11-based networks, numerous researchers have dealt with improving the energy efficiency acting on the various medium access control and physical layer mechanisms [7,8] or on network-wide algorithms, such as dynamic channel selection [9] and coordination functions [10]. Recent works have focused on resource allocation in Terahertz communications [11,12].

However, very few works can be found on the design of efficient reconfiguration algorithms to reduce the power consumption of the WLAN infrastructure-side when the load is scarce. In fact, most (or even all) of the currently deployed enterprise WLANs are continuously operated at full power; i.e., all Access Points (APs) are always turned on with the transmission power set to the maximum. This full-power operation produces a considerable energy wastage, because the same power is employed at the peak hours (e.g., 11 a.m. of weekdays) and during the off peak periods (e.g., nights and weekends).

In this work, we address the problem of saving energy during off-peak hours in enterprise WLANs, while preserving the same coverage and quality of service levels provided when the network is run at full power. To meet these goals, we operate at the network management level, where the available options are: (i) turn off some APs, and (ii) tune the level of the radiated—and hence consumed—power via the active APs. In order to determine the set of APs to switch off and the power levels to use on each AP, we act on the association between the APs and the user devices. By assigning the devices to the “proper” APs (the exact meaning of “proper” will be made explicit later on), we can create a subset of APs with no associated users, which can thus turned off. Further energy savings can be achieve by the powered-on APs by not transmitting at the maximum power, but still meeting the traffic demand of each associated user.

To this aim, we first define a general model of the WLAN, we then build a mathematical problem formulation, and we finally solve the problem to optimality by means of an efficient algorithm we developed for this purpose. Specifically, the model is used to take two kinds of decisions: (1) associate each user ( the term “user” should be taken in its most general sense; a more precise definition of what is going to be associated with the APs is given in Section 3) with one of the available APs, and (2) set the transmission power level of each AP.

In the first step of our work, our main goal was to model the WLAN in the most general form, i.e., without performing approximations or simplifications. Indeed, while such approximations and/or simplifications might, on the one hand, lead to a simpler mathematical programming problem (e.g., linear), on the other hand they might lead to solutions that are not applicable or unsatisfactory for the original problem.

The resulting WLAN model is then taken as the basis to formulate a mathematical programming problem. As it will emerge from Section 4, the formulated problem is a particular case of a broader class of location-design problems, where both location and capacity dimensioning decisions must be taken. It is therefore a NP-hard combinatorial program, but with the added difficulty of nonlinear constraints (in short, an integer nonlinear problem, INLP). Specifically, the nonlinearity is due to the nonlinear relation between the user assignment variables and the power level assignment variables.

Due to the problem complexity, its resolution to optimality by means of general purpose solvers is practically feasible for small scenarios only. Alternatively, iterative approximation algorithms (that converge to a solution that is close to optimal) or heuristics (that find a feasible solution in short times, but give no guarantees in terms of optimality, completeness, accuracy, or precision) can be employed. Indeed, the two latter approaches have been extensively adopted in the literature, both in the context of WLANs [13,14] and more in general, for cellular networks [15,16] and wireless sensor networks [17]. However, they provide just an arbitrarily “best” feasible solution to the addressed problem.

On our side, we devised a method and implemented an algorithm, named Benders’ decomposition-based algorithm (BDA), that yields the optimal solution to the original problem in a reasonably small computational time. To achieve this goal, we selected Benders’ decomposition (BD) technique [18], a rather popular approach to solve large mathematical programs with mixed (i.e., continuous and integer) variables. In particular, we adapted and customised BD to match the specific features of our integer nonlinear problem. Again, since we did not enforce any simplifications, the found solution is not only optimal for the mathematical programming problem, but also applicable to the real WLAN without performance degradation.

With regard to the user-AP association, it is worth recalling that, in IEEE 802.11-based systems, the user devices autonomously select the AP to associate with, generally by picking the AP from which they measure the highest received signal strength. As a consequence, it emerges the problem of how to put into practice the allocation computed by our approach. This practical problem, which has so far received scarce attention, is analysed in Section 7.

In summary, the main contributions of the paper can be condensed as follows. (1) We give a general characterisation of the WLAN and formulate a mathematical programming problem that keeps all these features without approximations. In particular, the formula that binds the AP transmission power to the available data rate at the user devices is left “open”, with the sole restriction of being non-decreasing. In this way, the mathematical problem formulation is disjointed from the specific data rate model, and hence can be widely applicable and re-usable. (2) Then, even though this leads to a NP-hard nonlinear combinatorial programming problem, we present an algorithm (named BDA) that is able to solve it to optimality in very short times. (3) By applying BDA to a series of realistic scenarios, we provide an insight on the impact of various parameters on the resulting allocation. Specifically, we quantify the possible energy savings and we show that the allocation strategy strongly depends on the problem geometry, that bigger scenarios (large number of APs) can lead to proportionally higher savings (hence the importance of having an algorithm that scales well), and that having many power levels at the APs can lead to further energy savings if properly allocated. (4) Finally, we discuss the deployment issues of BDA, providing reasons for the feasibility of the BDA approach even for currently deployed WLANs.

The rest of the paper is structured as follows. The next Section sketches the current state of the art in energy-efficient resource allocation in WLANs. Subsequently, Section 3 illustrates the analytical model of the WLAN. Then, Section 4 describes the mathematical programming formulation, and Section 5 shows the method we designed to solve it. The computational results are presented in Section 6. Some comments about the deployment of the optimal allocation are exposed in Section 7. Finally, the concluding remarks are drawn in Section 8.

## 2. Related Work

The default WiFi operation lets clients select the access point (AP) to connect to based on the received signal strength. This approach, known as client-driven user-AP association, has many shortcomings and does not allow the optimisation.

Software-Defined Networking (SDN) has changed the classical architecture of WLANs towards a more flexible approach in which a global view of the network is made available at a software-defined controller [19]. In this scenario, exploiting the Virtual Access Point (VAP) concept active user steering and seamless handover can be exploited to implement more sophisticated user association solutions [20,21].

The schemes for implementing user association can be centralised or distributed depending on the entity responsible for the association decision. The adoption of Software-Defined WLANs [19] gives more popularity to centralised schemes than distributed ones. The centralised schemes are based on the idea that a network controller collects network state information and optimises association considering different goals, such as aggregated throughput, user fairness [22], AP load balancing [23]. Furthermore, the centralised scheme can jointly perform different management functions, such as user association and channel assignment [24], and allows one to consider different aspects, such as the solution proposed in [25], which exploits link-layer multicasting to decide user-AP associations for providing simultaneous content distribution in a sustainable mode. In this paper, multicasting is not considered because multicasting refers to specific kinds of service.

These works consider architectural aspects and optimisation aimed to improve user experience and to increase resources utilisation. However, they do not study the energy savings in WLANs as this paper.

As already mentioned in the Introduction, despite the abundant literature on energy efficiency in wireless LANs [7,8,9,10,26], very few works exist that deal with a problem similar to the one faced here.

For example, Jardosh et al. [27] proposed a strategy to dynamically power on/off APs to follow the resource demand of the users. This approach has been translated into a working testbed, proving the feasibility of the idea and the related energy gains. However, the strategy is based on empirical considerations, has no guarantees of optimality, and is tested on a very small network.

On the other hand, Lorincz et al. [28] followed an optimisation approach based on an integer linear program (ILP). They employed an approximation of the date rate formula (based on few quantised coverage rings), and the ILP is tested on a single medium-sized scenario. The same authors confirmed the potential scalability issues of the ILP method solved through a general purpose solver, and consequently developed heuristic algorithms [13]. In addition, their problem is also different from ours as it targets a one month timespan with various traffic scenarios (thus adding further empirical constraints), whereas we focus on a single off-peak period (giving an exact solution).

Couto da Silva et al. [29] focused on a portion of a dense WLAN in which groups of identical APs are co-located in the same position. To decide the assignment of the users covered by more than one group, they exploited a queuing model, which, however, becomes impractical even when the coverage areas of more than two groups overlap.

Zhang et al. [30] investigated the power allocation and the placement of an energy-harvesting AP in a single cell WLAN with cooperative users. The goal of throughput maximisation, subject to energy constraints, is reached by means of an optimisation problem that is decomposed into two subproblems, which are solved to optimality. The problem definition, however, is quite distant from ours.

Wu et al. [31] studied the user association problem in green WLANs, to minimise the network’s energy consumption while satisfying time-varying traffic demands. They formulated an integer linear programming able to take into account both AP congestion avoidance and user migration constraints.

At last, we mention the work of Garcia-Saavedra et al. [14], who studied the trade-off between energy and throughput optimisation in case of heterogeneous user devices. Though presenting an exact analytical model, the authors simplified it due to its complexity. Anyhow, the addressed problem is different from ours, since it targets the energy consumption of the user devices in a single WLAN, rather than the WLAN infrastructure.

In fact, the client-side energy minimisation has been an active research topic, as shown for example in [32]. As already stated, we focus on the infrastructure-side, for which the problem is significantly different. This work is the extension of our previous paper [33].

In summary, none of these works combines optimality and fast solving times, while also considering the WLAN in its generality. Therefore, our approach can indeed be placed one step ahead of the current literature.

## 3. WLAN Model

Our focus is on already deployed WLANs, i.e., we do not solve the problem of choosing in which candidate sites the APs shall be deployed. We assume this has been done in a previous phase, for example on the basis on the peak traffic demand pattern. Due to the already widespread adoption of enterprise WLANs, this hypothesis matches quite well with reality. Additionally, it allows one to apply our method to existing networks, not just to the future ones. Accordingly, we use the terms “AP” and “site” interchangeably. Note, however, that the extension to the joint design and allocation problem (i.e., choosing also the locations of the APs), is, from the modelling perspective, trivial. As it will be clear later, it is sufficient to expand the set of APs by including also the potential deployment sites. Obviously, this inclusion would have an impact on the solving times of the algorithm (which, however, are rarely an issue for the planning phase), but it does not add any contribution to the problem formulation.

Let S be the set of deployed AP and U be the set of traffic nodes (TNs) to be assigned to the APs. Note that we refer to “traffic nodes” rather than to “users” or “user terminals”, and that the two concepts are slightly different. In particular, each TN represents the barycenter of an area that contains a quantum of demand. For example, a node may aggregate the traffic of all the user terminals present in a given office or room. This abstraction, named the demand node concept, is rather common in network design and resource allocation. It is worth remarking that the demand node concept allows one to build a stationary traffic model of a mobile population [34]. For this reason, we can assume that the TNs are static, and their positions are known. We also assume that all APs have the same characteristics, such as the power consumption and data rate behaviour.

**Figure 1:**
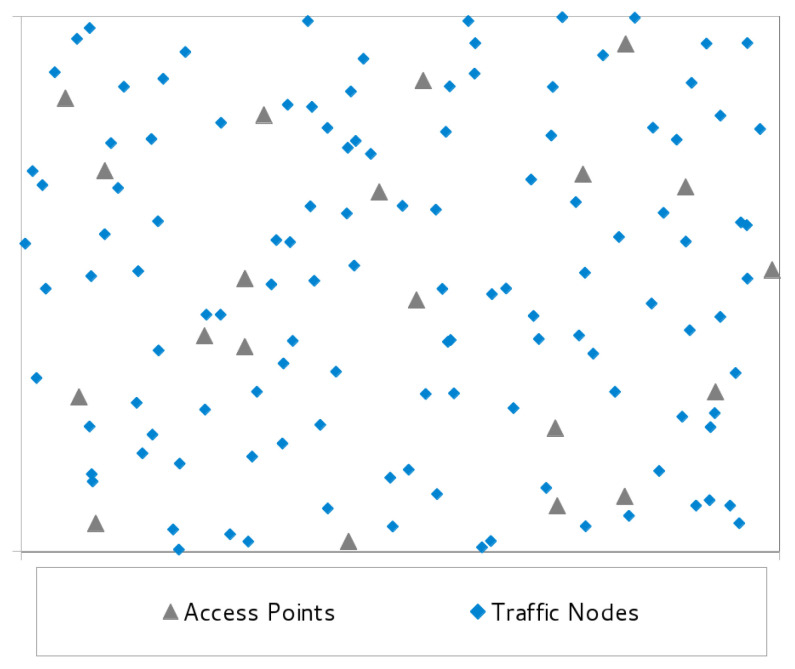
Sample instance topology of a wireless local area network.

This assumption is useful to keep the model description simple, but does not reduce its validity, since the extension to the heterogeneous case is straightforward. Figure 1 shows an example of WLAN topology with APs and TNs.

### 3.1. The Model of AP Power Consumption

The energy consumed by every AP can be divided into fixed and variable parts. The fixed part (say $P_{0}$) is bound to the mere fact that the device is powered on, and therefore encompasses AC/DC conversion, basic circuitry powering, dispersion, etc. The variable part essentially depends on the radiated power $p_{j}$. Hence, the power $P_{j}$ consumed by AP *j* can be expressed as:

(1) $$P_{j}=P_{0}+\eta \cdot p_{j},$$

where η is an efficiency factor (e.g., to account for the electrical model of the device).

### 3.2. Propagation and the Data Rate Model

The power received by node *i* from site *j* is determined through the radio propagation model. In the most general form, $p_{ij}^{R}=\alpha_{ij}\cdot p_{j}$, where $\alpha_{ij}\in \left[0,1\right]$ is the path loss between node *i* and site *j*. Once the physical properties of the problem (e.g., the position of nodes and sites, the frequency, and the attenuation factor) have been established, all $\alpha_{ij}$ can be computed and are a constant of the problem. Since we characterise the WLAN during a long-term period, we can safely assume that the channel is static. Short-scale channel fluctuations, which might be of interest for packet-level modelling, can be smoothed over the much longer reference time of our approach (hours), thereby allowing for the use of average $\alpha_{ij}$ values. Mid-scale fluctuations can be accounted for by means of an over-provisioning factor ρ (see Equation (10) later on), or with tools such as stochastic or robust optimisation (which can be an aspect for future investigation).

The received power $p_{ij}^{R}$ is used to compute the Signal-to-Noise Ratio (SNR) at node *i*: $\sigma_{ij}=p_{ij}^{R}/N_{i}$, where $N_{i}$ is the (thermal) noise at node *i*. We exclude the interference from our model because, by means of an accurate planning, it is possible to exploit the availability of the several non-overlapping channels ( for example, there are four non-overlapping channels in the 2.4 GHz band and up to 18 in the 5 GHz band in Europe for IEEE 802.11a/g) to achieve spatial reuse of the frequency. In this way, APs working on the same channel are sufficiently apart to not interfere with each other—or anyhow to make the interference very low. We also recall that the CSMA (carrier sense multiple access) scheme employed by IEEE 802.11 WLANs can greatly mitigate the impact of interference (possibly at the cost of reduced average capacity per AP—the ρ factor defined above may also be used for this purpose).

The SNR $\sigma_{ij}$ is used to derive the data rate $r_{ij}$ that is established between node *i* and site *j* (i.e., the capacity of link (i,j)). In general, this relationship can be arbitrarily complex, because it depends on various factors (e.g., current modulation and coding scheme, rate adaptation algorithms) in a nonlinear way. The only feature we make explicit is that $r_{ij}$ is a non-decreasing function of the radiated power $p_{j}$, which derives form both theoretical and practical evidences. To ease the discussion, we just write:

(2) $$r_{ij}=\psi_{ij}\left(p_{j}\right),$$

where $\psi_{ij}\left(\cdot \right)$ is a generic non-decreasing function.

### 3.3. The Traffic Model

Finally, we assume that every node *i* has a traffic demand $w_{i}$, which comprises both the uplink and the downlink traffic. Since we assumed a symmetric channel, there is no need to differentiate between the two directions because data transmissions occupy the channel in the same manner for both uplink and downlink.

From this description it is apparent that the WLAN model is very general, and the assumptions are limited to the absolutely essential. Consequently, our mathematical programming formulation is very general as well, and can thus be configured and applied to diverse scenarios and case studies.

## 4. Mathematical Problem Formulation

The objective of our problem is to minimise the total consumed power by both selecting the appropriate power level (PL) for each AP and assigning each TN to a site. To state the objective function, we define two families of binary decision variables:

- $x_{ij}$, if $x_{ij}=1$ then TN *i* is associated with AP *j*, otherwise TN *i* is not associated with AP *j*;
- $y_{jk}$, if $y_{jk}=1$ then AP *j* is using PL *k*, otherwise AP *j* is not using PL *k*.

Note that $y_{jk}$ is used to express $p_{j}$, the power radiated by site *j*:

(3) $$p_{j}=\sum_{k\in P}\;y_{jk}P_{k}^{T},$$

where we have assumed that $p_{j}$ can be represented by a discrete variable taking values in the set $\left\{P_{k}^{T}\right\},k\in P=\left\{1\ldots K\right\}$. Indeed, the majority of off-the-shelf APs have a set of preset power values to choose among (see, e.g., [35]).

The minimisation of the objective function can thus be stated as:

(4) $$z=\min\sum_{j\in S}P_{j}=\min\sum_{j\in S}\sum_{k\in P}\left(P_{0}+\eta P_{k}^{T}\right)y_{jk},$$

where we have grouped $P_{0}$ and $\eta P_{k}^{T}$ because, given that at most one PL is selected at each site *j*, there is no point in introducing further variables for the powering-on decisions (in other terms, if no $P_{k}^{T}$ is chosen, i.e., $y_{jk}=0\;\forall k$, the AP is automatically turned off). The constraints of the problem are the following:

(5) $$x_{ij}\in \left\{0,1\right\},\;\forall i\in U,\forall j\in S$$

(6) $$y_{jk}\in \left\{0,1\right\},\;\forall j\in S,\forall k\in P$$

(7) $$\sum_{j\in S}x_{ij}=1,\;\forall i\in U$$

(8) $$\sum_{k\in P}y_{jk}\leq 1,\;\forall j\in S$$

(9) $$x_{ij}\leq \sum_{k\in P}y_{jk},\;\forall i\in U,\forall j\in S$$

(10) $$\sum_{i\in U|r_{ij}>0}\frac{x_{ij}w_{i}}{r_{ij}}\leq \rho ,\;\forall j\in S$$

(11) $$x_{ij}=0,\;\forall \left(i,j\right)\in U\times S\;|\;r_{ij}=0,$$

where ρ∈(0,1] is a constant parameter. In detail, Equations (5) and (6) state the integrability of the decision variables, Equation (7) mandate that each TN must be assigned to exactly one AP, Equation (8) say that at most one PL can be selected for every AP, Equation (9) prescribe that TNs cannot be associated with powered-off APs, and Equation (10) are the capacity constraints, i.e., TNs must be assigned to APs so that each demand is met and the “usable” capacity of each AP is not exceeded. Finally, Equation (11) just state that a TN *i* cannot be assigned to an AP *j* if the rate on link (i,j) is zero.

### Comments on the Formulation

The formulation built by Equations (4)–(11) is nonlinear. Specifically, the nonlinearity originates from the dependence of $r_{ij}$ from the $y_{jk}$ variables, which comes from Equations (2) and (3). Therefore, Equation (10) are function of $x_{ij}/y_{jk}$, and thus we have a set of nonlinear constraints. Note, anyway, that the nonlinearity is not bound to the specific form of $\psi_{ij}\left(\cdot \right)$, but it simply derives from $r_{ij}$ being a function of $y_{jk}$. Even though $r_{ij}$ were a linear function of $y_{jk}$, Equation (10) would still be nonlinear. Obviously, if $r_{ij}$ were constant, the problem would become linear.

Some further comments about the constraints follow. By means of Equations (7) and (10), we aim at associating all TNs and accommodating the whole traffic demand. Thanks to the demand node abstraction, the association of all TNs might be equivalent, from a practical point of view, to covering the entire service area [34]. Obviously, to actually fulfil this goal, the position of the TNs must be accurately planned, and test nodes (i.e., TNs with zero, or almost-zero traffic) might also be accounted for. Such a planning is out of the scope of this work, but we nevertheless remark that our formulation and method are ready and can be used to achieve full area coverage.

Still about Equation (10), we inserted the term ρ to account for a guard interval on the APs airtime usage. Setting ρ<1 prevents the formulation from saturating the capacity of the APs, which might be desirable from a practical point of view (e.g., to avoid large packet losses and delays, and thus provide some form of QoS guarantees), but has no impact on the formulation (being just a parameter). Conversely, ρ=1 turns Equation (10) into their classical form.

## 5. Benders’ Decomposition-Based Algorithm (BDA)

As a consequence of the nonlinearity, the resolution of problem formulated in Equations (4)–(11) is hard both in theory and in practice. To tackle this problem, we used a Benders’ decomposition (BD) framework [18], which has been tailored to deal with the nonlinearities in our mathematical program. The Benders decomposition technique allows one to efficiently solve some mixed-integer non-linear problems, such as in [36], reducing a lot the computation time with respect to brute-force and greedy-like optimisation algorithms.

In general, BD proceeds by iteratively solving a master problem, M(y), and a subproblem, $S(x,\bar{y})$. The purpose of M(y), which has the *y* variables only and is solved to optimality, is to fix *y* to values $\bar{y}$; then, $S(x,\bar{y})$, which has the *x* variables only, is used to verify the feasibility of the $\bar{y}$ assignment. The advantage of BD is that the resolution of both M(y) and $S(x,\bar{y})$ is much simpler than the original problem.

In our case, we defined M(y) as Equations (4), (6), (8), and:

(12) $$\sum_{j\in S}\sum_{k\in P}r_{ij}^{k}\;y_{jk}\geq w_{i},\;\forall i\in U,$$

where $r_{ij}^{k}=r_{ij}\left(P_{k}^{T}\right)$. In addition, a set of inequalities, called Benders’ feasibility cuts, is dynamically generated and added at each iteration.

Inequalities in Equation (12) just state that, for the problem to be feasible, there must be enough capacity in the WLAN to satisfy each demand. The $r_{ij}^{k}$ are still computed by means of $\psi (p_{j})$, but they are constant values because $p_{j}$ is fixed to the $P_{k}^{T}$ values, and consequently, $r_{ij}^{k}$ are no longer a function of $y_{jk}$, so that M(y) is linear.

Equation (12) are thus a weaker but linear form of capacity constraints. This linear constraints allowed us to retain the nonlinear relation between $r_{ij}$ and $p_{j}$, and at the same time build a linear problem which can be efficiently solved. The actual enforcement of Equation (10) is verified later by means of $S(x,\bar{y})$.

To formulate $S(x,\bar{y})$, let $\bar{S}\subseteq S$ be the set of APs that are powered on as per the solution of M(y), and ${\bar{r}}_{ij}$ be the capacity of link (i,j) as it results from Equations (2) and (3) once $y_{jk}={\bar{y}}_{jk}$. Then, $S(x,\bar{y})$ has the following form:

(13) $$x_{ij}\in \left\{0,1\right\},\;\forall i\in U,\forall j\in \bar{S},$$

(14) $$\sum_{j\in \bar{S}|{\bar{r}}_{ij}>0}x_{ij}=1,\;\forall i\in U,$$

(15) $$\sum_{i\in U|{\bar{r}}_{ij}>0}\frac{x_{ij}w_{i}}{{\bar{r}}_{ij}}\leq \rho ,\;\forall j\in \bar{S}$$

(16) $$x_{ij}=0,\;\forall \left(i,j\right)\in U\times \bar{S}\;|\;{\bar{r}}_{ij}=0.$$

Note that Equation (15) are linear constraints, because, being ${\bar{r}}_{ij}$ fixed, they depend solely on the $x_{ij}$.

We then check for the feasibility of $S(x,\bar{y})$, and if $S(x,\bar{y})$ is feasible, $\bar{y}$ is an optimal solution and we can stop the algorithm. Otherwise, if $S(x,\bar{y})$ is unfeasible, we must add a feasibility cut to remove the unfeasible solution $\bar{y}$ from M(y), and solve M(y) again.

A simple version of Benders’ feasibility cut can be written as:

(17) $$\sum_{j\in S|{\bar{p}}_{j}=0}\sum_{k\in P}y_{jk}+\sum_{j\in S|{\bar{p}}_{j}>0}\sum_{k\in P|P_{k}^{T}>{\bar{p}}_{j}}y_{jk}\geq 1,$$

where ${\bar{p}}_{j}=\sum_{k\in P}\;{\bar{y}}_{jk}P_{k}^{T}$. This cut can be identified by remarking that an unfeasible $S(x,\bar{y})$ implies that $\bar{y}$ does not provide enough capacity to satisfy the demands. Therefore, we must either use more APs (as determined by the first term in Equation (17)) or raise the PL of at least one AP (second term). Note that the latter implies having greater $r_{ij}$’s, and thus more capacity, because of $\psi_{ij}\left(\cdot \right)$ being non-decreasing.

The following theorem proves the convergence of BDA to the optimal solution of problem Equations (4)–(11).

**Theorem** **1.** *If the problem in Equations (4)–(11) is feasible, then BDA determines an optimal solution to it.*

**Proof.**  At each iteration of BDA, the master problem M(y) defines a (progressively tighter) relaxation of formulation in Equations (4)–(11). Therefore, any optimal solution $\bar{y}$ of M(y) yields a lower bound $z^{l}$ to the optimal objective function value *z*. On the other hand, if $S(x,\bar{y})$ is feasible, then $\bar{y}$ is a feasible solution, and the corresponding consumed power provides an upper bound $z^{u}$ to *z* (because any feasible solution provides an upper bound to the optimal objective function value). However, the value of Equation (4) when $y=\bar{y}$ is exactly $z^{l}$, as determined by the solution of M(y). Therefore, since the lower bound coincides with the upper bound, the optimality of the $\bar{y}$ assignment is certified.

Otherwise, i.e., if $S(x,\bar{y})$ is unfeasible, then no feasible *x* solution exists for the current assignment $\bar{y}$. In such a case, BDA adds the feasibility cut (17) to M(y), and iterates by solving the enhanced relaxation. Observe that adding cut (17) avoids the future generation of the unfeasible assignment $\bar{y}$, and it is correct since $\bar{y}$, being unfeasible, can not be part of any optimal solution to problem in Equations (4)–(11). Since the number of possible $\bar{y}$ assignments is finite, it follows that after a finite number of additions of cuts (17), BDA must end with an optimal solution to the problem in Equations (4)–(11). □

As a final remark, note that the form of the function $\psi_{ij}\left(\cdot \right)$ has no (or very little) impact on the algorithm, because the rates $r_{ij}$ are used either as $r_{ij}^{k}$ or as ${\bar{r}}_{ij}$. In both cases they are samples of $\psi_{ij}\left(\cdot \right)$ in some given points, and therefore it is not relevant, from the BDA point of view, whether $\psi_{ij}\left(\cdot \right)$ is linear or arbitrarily complex (provided that, as already stated in Section 3, it is non-decreasing). Note that if we have a system able to estimate the date rate, these estimation can be profitably used.

Additionally, note that neither BDA, nor the mathematical programming formulation, make assumptions on the amount of traffic they deal with. In other terms, both the formulation and the algorithm can be applied to any traffic scenario. Of course, the application to the off-peak hours, which is the topic of this work, is of particular interest because, as it will be shown in the following, it allows one to achieve considerable energy savings.

**Figure 2:**
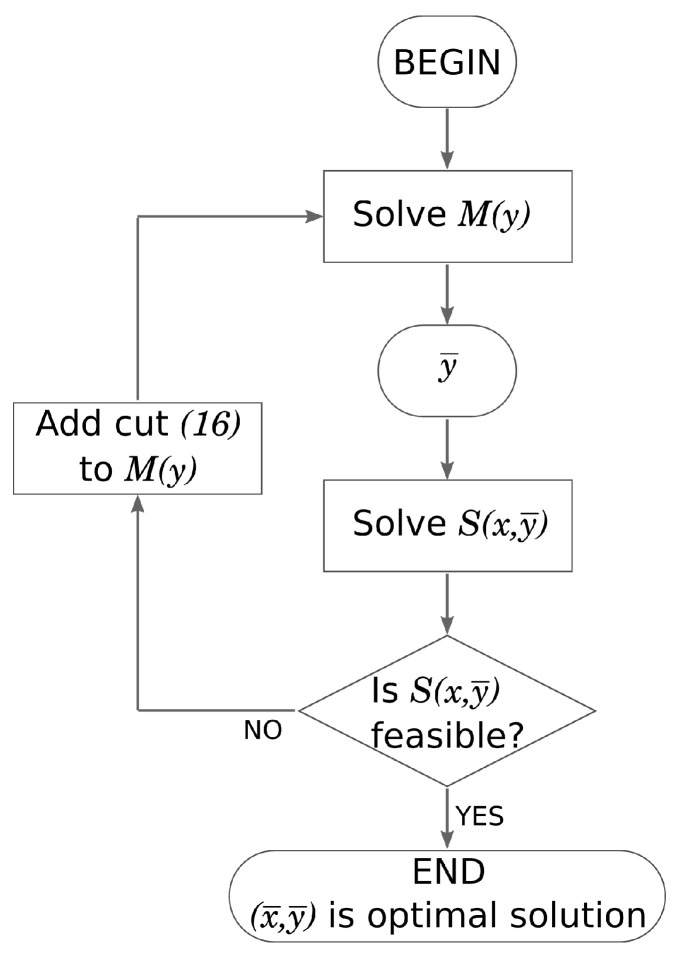
Flow chart of Benders’ decomposition-based algorithm.

BDA has been implemented in C++. Figure 2 illustrates the flow chart of BDA. In our implementation, the blocks “Solve M(y)” and “Solve $S(x,\bar{y})$” has been handled, for convenience, by means of the IBM ILOG CPLEX solver version 12.5. However, any other ILP solver can be employed for this purpose.

## 6. Performance Evaluation

### 6.1. Test Scenarios

We assessed the performance of BDA for a series of nine scenarios, whose features have been extracted from real life measurement campaigns in corporate environments [27,37]. The number of APs, TNs, and PLs, the average off-peak traffic demand of each node ($\bar{w}$), and the mean distance among the APs (*D*; a larger *D* implies a sparser network) are reported in Table 1. In table, the parameters that are variated with respect to the reference scenario R are highlighted in bold.

The off-peak traffic has been set to roughly one tenth of the peak one. This latter has been computed by solving a simple ILP in which all APs are active and operate at the maximum power ($y_{jk}=1$ if $k=k_{max}$, 0 otherwise, with $k_{max}$ being the index of $P_{max}^{T}$), and then by maximising the network load ($\sum_{i\in U}w_{i}$). The actual demand $w_{i}$ assigned to each TN is a random value extracted from a uniform distribution whose extremes are [$0.9\;\bar{w}$, $1.1\;\bar{w}$] (see the computed $\bar{w}$ in Table 1).

The first scenario is used as the reference one: starting from it, we have changed the other parameters (in bold in Table 1) in order to estimate their impact on the algorithm performance. All variations have been analysed at both 21 and 42 m (to disambiguate, a “@ 21m” or “@ 42m” will be appended to the scenario name to identify the value of *D* where necessary).

For all scenarios ρ was set to 0.9. This value serves just as a case study, being the choice of ρ dependent on the degree of saturation at which the administrator wants the network to operate. In other terms, ρ can be used to tune the working point of the network, but we recall that it is has no impact on the behaviour of the allocation algorithm.

**Figure 3:**
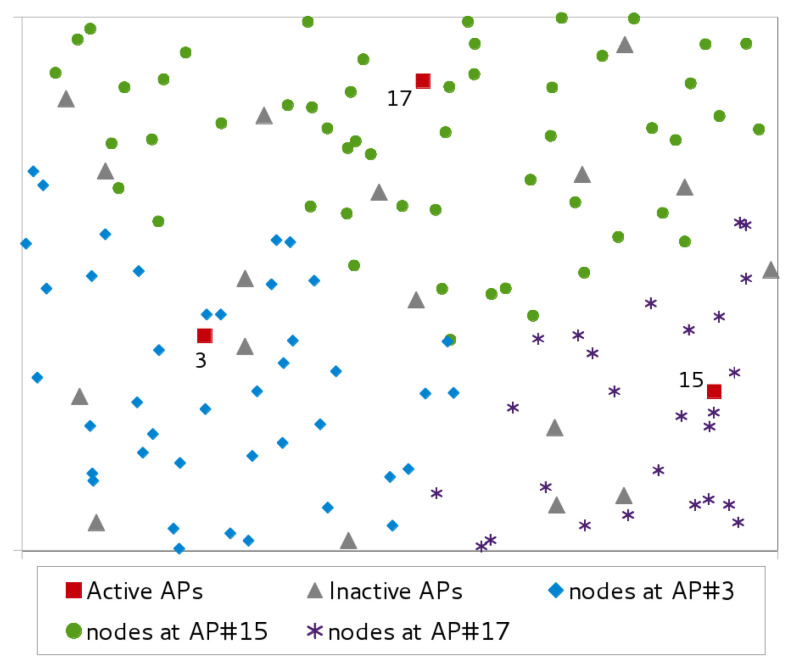
Sample assignment of traffic nodes to access points.

For each scenario, we generated and solved twenty instances. The positions of the APs and TNs in the test area of each instance have been randomly determined. However, to guarantee a minimum amount of rationality, we have divided the test field into a regular grid of |S| squares. The APs are placed one per square, with the coordinates chosen randomly within the square. The set of nodes is also split into |S| subsets, and the nodes of each subset are randomly spread over each square. This strategy ensures enough uniformity in the placement of TNs and APs to mimic a corporate scenario and to avoid heavily unbalanced instances. An example of the topology produced by our instance generator war reported in Figure 1. Figure 3 is the solution found by BDA. The figure shows active and inactive APs, but for the sake of simplicity the power level of the active APs is not reported.

For the signal propagation we employed a simplified version the COST-231 multi-wall path loss model for indoor, non-LOS environments [38]. The path loss α (in dB) at distance *d* is computed according to:

(18) $$\alpha =L_{0}+L_{c}+10n\log_{10}\frac{d}{d_{0}}+N_{W}L_{W}+N_{C}L_{C},$$

where $L_{0}$ is the reference path loss, measured at the reference distance $d_{0}$, $L_{c}$ is a constant loss (arising from multi-wall curve fitting), *n* is the path loss exponent, $N_{W}$ and $N_{C}$ are the number of penetrated walls and columns, and $L_{W}$ and $L_{C}$ are the losses of walls and columns.

To set the values of the above parameters, we referred to the several measurement works on this model [39,40]. However, since the reported numbers differ substantially, we opted for using a series of middle values, which are shown in Table 2. To compute $N_{W}$ and $N_{C}$ we measured the distance between two walls/columns in our Department, resulting in an average separation of 8 and 20 i.e., respectively. A 3 dBi omnidirectional antenna has been assumed to be mounted at the APs.

Then, we had to define the ψ(·) function that determines the rates $r_{ij}$. As reported in several experimental studies, such as [41], it is often possible to bind the SNR expressed in dB ($\sigma_{ij}^{\left[dB\right]}=p_{j}^{\left[dB\right]}+\alpha_{ij}^{\left[dB\right]}-{N_{i}}^{\left[dB\right]}$) to the data rate by means of a linear function. In detail, $r_{ij}$ will be a function of $\beta \cdot \sigma_{ij}^{\left[dB\right]}+\delta$, where β and δ are two suitable “linearisation” factors. A further aspect to be considered is that, when the received power $p_{ij}^{R}$ falls below a given sensitivity threshold γ, we must assume $r_{ij}=0$. Similarly, we must also cap $r_{ij}$ to the maximum rate achievable by the physical link, say $r_{max}$. Just to mention an example, the IEEE 802.11g standard cannot deliver more than 54 Mbps, no matter how high the SNR is. Thus, we can summarise the relationship between $r_{ij}$ and $p_{j}$ with this unique nonlinear (and non-decreasing) expression:

(19) $$r_{ij}=\left\{\begin{array}{c} \min\left\{\beta \cdot \sigma_{ij}^{\left[dB\right]}+\delta ,r_{max}\right\},\;\mathrm{if}\;p_{ij}^{R}>\gamma_{P},\\ 0,\;\mathrm{otherwise}. \end{array}\right.$$

The thermal noise at the nodes, $N_{i}$, was set to −125 dB, the sensitivity threshold γ was assumed to be −121 dB [35], and the rate linearisation factors were fixed according to the work of Zhang et al. [41], resulting in β=1.76 and δ=−7.48.

To complete the parameter list, we set the consumption figures of the APs according to [35]: $P_{0}=12\;W$, η=30, and $P_{k}^{T}$ taking values in the range from $P_{max}^{T}$ = 0.1 W to $P_{min}^{T}={\left(\frac{1}{2}\right)}^{|P|-1}\;P_{max}^{T}$, with

(20) $$P_{k+1}^{T}=\frac{1}{2}\;P_{k}^{T},\;k=1,\ldots ,K-1,$$

where, clearly, $P_{1}^{T}=P_{max}^{T}$ and $P_{K}^{T}=P_{min}^{T}$. Hence, according to Equation (1), the maximum AP power consumption is $P_{j}=P_{0}+\eta \;P_{max}^{T}=12\;W+3\;W=15\;W$.

### 6.2. Computational Results

For each scenario we collected various statistics about the performance (output) of our method. Table 3 and Table 4 report the average number of powered-on APs, the average power consumption, the gain with respect to the peak-hour case, the average number of TNs assigned to each powered-on AP (TN/AP), and the average load (airtime) per AP for the two sets of scenarios.

From a comparison with Table 1, it emerges that, for all scenarios at *D* = 21 m, a very small fraction of the APs is powered on. Having few powered-on APs leads to considerable energy savings, which span between 86.4 and 90.4 percent. Such gains are allowed by the relatively dense distribution of the nodes and APs, and obviously, by the relatively low traffic demand. In fact, however, the former factor is the dominating one.

The average power gain in the scenarios at *D* = 42 m is sensibly lower (61.6% on average), the number of TNs per AP and the airtime are much smaller, and the number of active APs is drastically increased. The main cause is the expanded inter-AP distance, which requires more APs to be powered on to cover all TNs and thus determines higher consumptions, smaller TN/AP ratios and lower airtimes.

More in-depth considerations can be extrapolated by analysing how the problem behaves in relation to the parameter changes.

#### 6.2.1. The Effect of the Number of Access Points

Starting with the size of the scenario (i.e., the number of APs, |S|), the output data are almost linearly dependent on the input data. Reducing |S| by 2.5 (scenario A1, |S| = 20), roughly halves the network power consumption and the number of powered-on APs. The power gain is also slightly reduced (in fact, it is the lowest among all scenarios), as are TN/AP and the average airtime. On the other hand, when |S| is doubled (scenario A2, |S| = 100), the power consumption and the number of active APs are slightly less than doubled, while the other figures have smaller variations. Therefore, the bigger the scenario size |S|, the better the improvement that the optimal allocation can bring, because the necessary network resources grow less than linearly with the number of APs.

The same considerations can be applied almost unchanged to the case *D* = 42 m. The only difference is that for the scenarios A*@42m there are smaller changes in the TN/AP ratio and average airtime than A*@21m. Conversely, the variation in the number of active APs and power consumption follows more closely the variation of |S|. These data indicate that the sparser scenarios leave less opportunity to exploit the “economies of scale”.

#### 6.2.2. The Effect of the TN/AP Ratio

Changes in the input TN/AP ratio also determine changes in the allocation pattern. In detail, when the TN/AP ratio is low (scenarios B1), there is a small decrease in the amount of allocated resources with respect to the reference scenario, and thus a further energy gain improvement. Similarly, when the TN/AP ratio is high (scenarios B2), there is a small increase in the amount of allocated resources, and a marginal reduction in the energy efficiency. Thesefore, in this case too, the output data moves more slowly than the input one, and also that a decrease in the number of TNs permits a sensible energy saving. The only figures that have a conspicuous jump are those about TN/AP and airtime, which directly reflect the modification of the input data.

#### 6.2.3. The Effect of the Number of Power Levels

**Figure 4:**
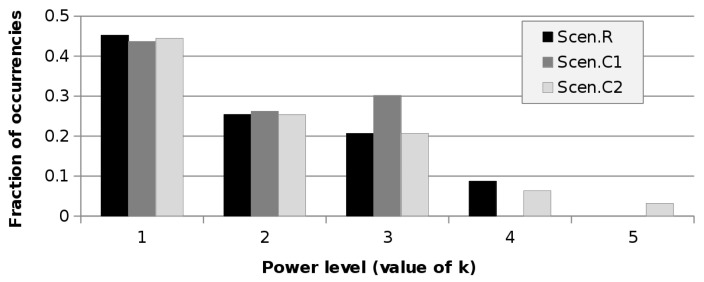
Occurrences of the power levels as a function of the test scenario for the case *D* = 21 m.

The impact of the number of PLs available at the APs is quite limited. Indeed, the values of scenarios C1 and C2 are almost the same as those of R, for both *D* = 21 m and *D* = 42 m. Figure 4 shows the occurrence of the various PLs chosen by the optimisation algorithm for scenarios R, C1, and C2 at 21 m. Smaller *k* means greater $P_{k}^{T}$ (as per Equation (20)). It can be seen that the first PL is largely the most used. The changes among the scenarios are limited and related to the higher *k* values. In scenario C1, the third $P_{k}^{T}$ is selected more often than in scenarios R and C2 as the consequence of having only three levels available at the APs. However, this different allocation has a very small impact on the power consumption, which is almost the same as R. The fifth PL, introduced in scenario C2, is selected just four times. Such a low utilisation of the additional power level indicates that there is no point in having many PLs at the APs, since the lowest ones are scarcely useful (at least in this context).

**Figure 5:**
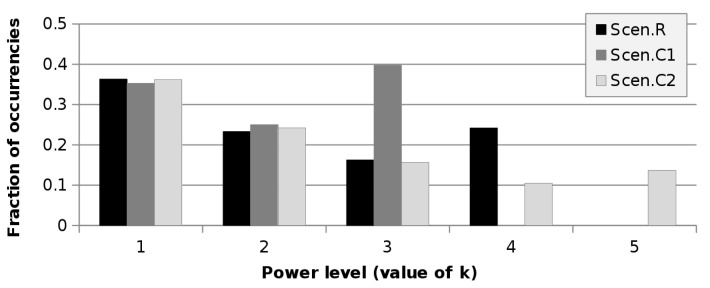
Occurrences of the power levels as a function of the test scenario for the case *D* = 42 m.

The same analysis has been performed also at double AP distance, i.e., *D* = 42 m. In this case the differences among the scenarios emerge more sharply, as illustrated in Figure 5. The reduction in the number of available PLs (scenario C1) implies a sensible shift in the allocation pattern. The lowest level is chosen more often, and to compensate, the highest PL is used a bit less. All in all, however, scenario C1 leads to a slightly higher network energy consumption (see Table 4). Conversely, a larger number of choices (scenario C2) allows a marginal gain improvement thanks to the use of the last PL.

The different behaviour between the scenarios at *D* = 21 m and at *D* = 42 m can be ascribed to the fact that in the latter case the APs are less loaded and serve less TNs. Thus, they can diminish the power emissions without compromising their performance. Additionally, note that both scenarios C1 and C2 obtain a reduction in the average airtime with respect to R. However, the former (C1) cannot be regarded as an improvement, because it is tied, in scenario C1@42m, to an increased power usage. On the contrary the latter is a real optimisation, because both the overall power and the airtime have been reduced. Therefore, when the network is “sparse”, the availability of a bigger set of PLs is indeed beneficial to the network optimisation problem.

#### 6.2.4. The Effect of the Traffic Load

The sensitivity of the problem to a variation in the demand is studied by means of scenarios D1 and D2. Again, there are very subtle differences with scenario R. In scenario D1, even though the traffic is notably reduced, it is just possible to file the power consumption a little, because the network is already working close to the “minimal configuration state”. In other terms, any other solution with a smaller energy footprint (either less powered-on APs or smaller power levels) would result in the disconnection of one or more TNs from the WLAN.

In scenario D2, since the network is in a sufficiently unloaded state, there are free resources to accommodate the added traffic without substantially changing the allocation. However, note that in D2@21m the WLAN is working almost at its saturation point (being the average airtime close to the 0.9 limit set for ρ). Therefore, even small increases in the offered load would force the solution to employ more resources. Indeed, we have verified that even setting $\bar{w}$ = 675 kbps yielded 6 active APs, with a power consumption of 83.8 W, and an average airtime of 88.2%.

#### 6.2.5. Solving Time

The last aspect to evaluate is the computational effort required by BDA. Table 5 reports the average CPU time, in seconds, for executing BDA on a PC equipped with a 2.27 GHz 64-bit processor. We remark that the CPU time is not dependent on the number of CPU cores. Thus, if you have a *n*-core processor, the real elapsed time is roughly *n* times smaller than the CPU time. It appears that in many cases BDA takes just a bunch of seconds to calculate the optimal allocation. Even in the most complex A2 scenario, BDA yields the solution in less than 5 min. The solving times for D=42m are generally smaller than D=21m because the sparser scenario implies that less TN-AP connections must be assessed (i.e., more $r_{ij}=0$ cases), and thus the complexity of the problem is lower.

These numbers clearly makes BDA suitable for the off-line allocation of the resources that can be performed on a weekly, daily, or hourly basis (depending on the assumptions on the peak/off-peak periods). For some (usually small) scenarios, given the very low running times, it is not to be excluded a possible application of BDA for quasi-real-time re-allocation of the resources in case of unexpected but stationary traffic changes.

### 6.3. A Comparison with a Similar Method

As outlined in the Introduction, the approach proposed by Lorincz et al. [28] is probably the most similar to ours that can be currently found in the literature. We have therefore performed a comparison between the problem formulation, named ME (model energy as in [28]) and our algorithm (BDA), in terms of solving time and overall power consumption.

Note however, that ME has a slightly different target than BDA. In fact, the goal of ME is to minimise the energy consumption over a given time period, whereas BDA optimises the instantaneous power consumption. Additionally, ME uses a different data rate model. Therefore, to enable a comparison we had to partially amend and simplify ME. Obviously, this operation led to a problem formulation, say RME (reduced ME), that was not built for our scenario, and thus the comparison we provide can serve as a further information, but is not to be meant as an absolute claim of superiority of BDA over the original ME.

In brief, Lorincz’s ILP formulation (ME) derives from a WLAN model which is very similar to the one in Section 3. However, when stating the relationship between the data rate $r_{ij}$ and the transmitted power $p_{j}$, Lorincz et al. discretised the coverage areas of the APs and the data rates between the APs and the TNs. In detail, every AP coverage area is split into a number of concentric rings of radius $d_{r}$, with r∈{1,2,3} being the ring index. All TNs in the same ring have the same rate, i.e., $r_{ij}\in \left\{{\bar{R}}_{kr}\right\}$, where ${\bar{R}}_{kr}$ is the average data rate for ring *r* when the AP operates at the *k*-th power level.

Taking advantage of this approximation, ME is then described by Equations (6)–(17) in [28]. As already outlined, however, we had to exclude some features that extends beyond the scope of our work. Specifically, we have removed the dependence on the time index *t*, the best-power selection constraints (10), and the excessive number constraints (13). The resulting RME formulation has then been implemented in AMPL and fed to the CPLEX solver.

As a final step before starting the computational analysis, we assigned the values to $d_{r}$ and ${\bar{R}}_{kr}$ so as to make RME input data coherent with BDA. To this aim, we employed the propagation model described in Section 3, which has been used for BDA too. Given that the maximum distance at which $r_{ij}>0$ is 40 m, we set $d_{1}$ = 14 m, $d_{2}$ = 27 m, and $d_{3}$ = 40 m. Then, the average data rates have been determined by considering a TN placed roughly in the middle of each ring (i.e., at 7.5, 20.5, and 33.5 m from the AP). The values of the various ${\bar{R}}_{kr}$ are reported in Table 6. Note that in the third ring the available data rate is zero when the lowest power levels are used, because the received signal is below the sensitivity threshold.

All instances have been solved on a PC equipped with a processor operating at 2.27 GHz with 24 GB of RAM. Table 7 reports the outcome of the comparison on the set of scenarios at *D* = 21 m (first nine lines), plus the reference scenario at *D* = 42 m. The first column reports the average solving time (in seconds) for BDA, while the successive columns refers to RME. In detail, they show the average solving time of RME, the power consumption of the RME solution, the amount of missed power saving of RME with respect to the optimal solution found by BDA (loss [%]), and the percentage of RME solutions that are actually unfeasible (U.I. [%]). As for the U.I. parameter, it has been computed by verifying the compliance of the RME solution to constraints (10). In other terms, we verified that the allocation yielded by RME is feasible when the actual data rates are employed. The figures in the block of columns marked “RME, $d_{3}$ = 40 m” refer to the computation of $d_{r}$ and ${\bar{R}}_{kr}$ as illustrated before. The purpose of the second block of columns, labelled “RME, $d_{3}$ = 24 m”, will be explained later. A further note about this table is that, since we noticed that RME took very long times in solving the various instances, we set a CPU time limit that is the greatest between 3600 s and twice the solving time of BDA. When the limit is exceeded, we recorded the best solution found so far.

The numbers in Table 7 are impressively in favour of BDA. Starting with the scenarios @21m with $d_{3}$ = 40 m, we can see that the solving times of RME are in some cases even three orders of magnitude greater than BDA. In addition, RME causes, on average, a 12.6% more consumption than BDA. These facts already give a remarkable indication of the usefulness of Benders’ decomposition approach.

A closer look at the table allows one to discover that in two scenarios RME appears to be marginally better than BDA (i.e., B1@21m and D1@21m). However, these gains cannot be deemed real, because, as the U.I. column shows, there is a high percentage of unfeasible solutions. Indeed, more than half the solutions yielded by RME can not be put into practice. The reason is that TNs that are close to the outer ring borders are assigned to the AP assuming they can use the average data rate of that ring. In practice, however, their real data rate might be far smaller than that, especially if the power level of the AP is not set to the maximum. For example, we have observed that in the second instance of scenario R@21m, RME assigns TN 110 to AP 28, setting the power level of AP 28 to k=2. Since the distance between TN 110 and AP 28 is 36 m, according to Table 6 it should be $r_{110,28}={\bar{R}}_{2,3}$ = 6.7 Mbps. However, according to the actual rate formula in Equation (19), it is $r_{110,28}$ = 0.024 Mbps, which is even less than the demand of TN 110 (159 kbps). It is therefore impossible to put the solution provided by RME into practice. Unfortunately, this circumstance occurs quite often, which leads to a high percentage of U.I. for RME.

A possible countermeasure could be to make a more conservative assumption in the definition of the ring boundaries and the associated data rates. Hence, we have run a second set of experiments assuming that the ring diameter is defined by employing the lowest power level at the APs, and that the data rates are computed on the external ring boundaries, rather than in the middle. This assumption leads to smaller rings, with $d_{3}$ = 24 m, and ensures that the actual rate of all TNs is no smaller than ${\bar{R}}_{kr}$, thereby guaranteeing the feasibility of the RME solutions. A side effect of these assumptions is that the performance of RME in terms of power saving is definitely worse.

Indeed, the “RME, $d_{3}$ = 24 m” columns of Table 7 prove our arguments. The solving times of RME are better than in the previous case – but still very high, whereas the power consumption of the RME solution has more than doubled.

The last row Table 7 shows the performance of both BDA and RME when applied to the sparser scenarios. We have reported just the reference one because the results for the others are very similar to it, and have the same general behaviour observed in Table 4 and in the first nine lines of Table 7. The computational times of BDA and RME are now comparable, with a small advantage for RME. However, the vast majority of the RME solutions are unfeasible (U.I. = 0.9). Furthermore, when applying the conservative estimate of $d_{r}$ and ${\bar{R}}_{kr}$, RME is never able to find a solution, because shrinking the coverage rings while having more distant APs leaves several TNs out of the coverage of the APs.

In summary, RME is often order of magnitude slower, and its solutions are either unfeasible or far less energy efficient than BDA. Therefore, even the use of heuristics to solve RME in faster times cannot be regarded as advantageous, because BDA already provides an optimal solution in acceptable times (less than 5 min in the most unfavourable case, just a few seconds in most scenarios).

As a final remark, it is worth noting that, besides the just analysed quantitative and qualitative differences, our work differs from Lorincz’s also in the way the optimal resource allocation problem is dealt with. In general, the first step in solving an optimisation problem is to characterise and transform it into a mathematical problem formulation. Then, the problem is solved by means of suitable tools and methods. In our context, the energy efficient WLAN resource allocation problem translates to the problem formulation described in Section 4. This problem formulation is quite general, and includes the data rate model defined in Section 3. In this phase, however, Lorincz et al. performed some approximations (the use of discretised coverage rings and average data rates) that allowed them to define an integer linear program. Such ILP has the advantage to be linear, and hence directly manageable by a solver such as CPLEX–mind, though that linearity alone does not give any guarantees in terms of tractability: problem formulation in Equations (6)–(17) in [28] is, in fact, NP-hard. As a consequence, while the found solutions are optimal for the problem itself, they might not be feasible for the original problem, as we have just shown. On our side, we did not enforce any simplifications, but developed an algorithm that efficiently solves to optimality the general problem. Therefore, all our solutions are always feasible (and optimal) for the original problem.

## 7. Implementation Considerations

As briefly outlined in the Introduction, the target of our work is the typical infrastructured enterprise WLAN. A series of APs is deployed in one or more buildings, or even in open areas (e.g., campuses), and every AP is connected to a central controller. Modern commercial controllers usually allocate the radio resources, configure the security settings on the APs, handle user authentication, manage the quality of the service, etc.

From our perspective, however, the controller should also be in charge of an extra task: computing the energy-saving allocation (i.e., executing BDA) and then putting it into effect. To this purpose, three aspects should be analysed: (i) how the controller gathers the necessary information from the network, (ii) whether the controller has sufficient resources to run the algorithm, and (iii) how the controller can enforce the allocation yielded by the optimisation algorithm.

As for the first point, the information we need is essentially the set of available rates $r_{ij}^{k}$, and the traffic demand of the nodes ($w_{i}$). Both sets of data can be obtained in a relatively easy way. The link quality can be assessed either by the various APs the users associate with or by means of the radio resource measurement facility introduced with the IEEE 802.11k standard. The traffic demand can be derived from a historical analysis or by exploiting the available traffic pattern models.

Regarding (Section 7), consider that substantial changes in the traffic pattern are quite infrequent, usually just the peak/off-peak scenarios are accounted for, and at most a few scenarios per day might be meaningful (see, e.g., [28]). Consequently, the controller would have some hours to compute the allocation. We have seen in the previous Section that the running times of BDA are relatively short (tens of seconds). Therefore, even considering a moderately powerful hardware, there seems to be adequate room to run BDA to completion.

Finally, putting into practice the optimal allocation is definitely more difficult. While turning the APs on/off and setting their PLs is a rather straightforward task, forcing the users to associate with a given AP is not trivial, because in IEEE 802.11-based WLAN the association procedure is client-based, and the infrastructure has hardly any control with which AP each terminal chooses to associate.

To date, however, some methods have been developed to make this operation feasible. At first, the recently ratified IEEE 802.11v standard allows the APs to request the users to transition to a specific AP, or to indicate a set of preferred APs. In the absence of that, it is still possible to use blacklists at the APs, as performed by Jardosh et al. [27]. Lastly, programmable network frameworks such as Odin [42] can create a series of user-specific virtual APs, by means of which it is possible to force the client stations to associate with a given AP. Recently, the SDN architecture considerably reduced the implementation difficulties and the experimental analysis of centralised optimisation schemes. Indeed, in [25] the authors show the experimental implementation of the user association solution of an optimisation problem, using the open-source SDN-based platform 5G-EmPOWER [20].

## 8. Conclusions

In this paper, we have presented a general mathematical programming problem and an exact and fast solving algorithm (BDA) for the optimisation of the energy consumption of enterprise WLANs during off-peak hours. The advantages of the approach are twofold. First, having disjointed the data rate model from the optimisation formulation, the latter can be used with arbitrarily complex data rate functions. Secondly, BDA efficiently solves the nonlinear problem formulation to optimality, for whatever non-decreasing data rate function. The computational analysis, in a series of realistic scenarios, proved the quality of BDA in terms of both achievable power savings and solving time.

Furthermore, the analysis shed light on some interesting aspects of the energy-efficient planning. In particular, on the basis of the power model of currently deployed APs, the solution of the green WLAN problem tends to apply the “consolidation” approach to the AP resource; i.e., the optimal strategy is to turn off as many APs as possible. The remaining APs are operated at the lowest power level, guaranteeing connectivity (and service) to all nodes. The availability of many power levels can thus be a further beneficial aspect, especially in circumstances such as sparse and lightly loaded networks.

References yes

## Figures and Tables

**Table 1:** Parameter values for the test scenarios.

Scenario	|S|	|U|	|U|/|S|	|P|	$\bar{w}$ [kbps]	*D* [m]
R	50	300	6	4	450	21/42
A1	**20**	**120**	6	4	450	21/42
A2	**100**	**600**	6	4	450	21/42
B1	50	150	**3**	4	450	21/42
B2	50	450	**9**	4	450	21/42
C1	50	300	6	**3**	450	21/42
C2	50	300	6	**5**	450	21/42
D1	50	300	6	4	**300**	21/42
D2	50	300	6	4	**600**	21/42

**Table 2:** Values for the employed path loss model.

Parameter	Value
path loss exponent (*n*)	2.34
reference distance ($d_{0}$)	1 m
reference path loss ($L_{0}$)	40.1 dB
constant loss ($L_{c}$)	14.2 dB
wall loss ($L_{W}$)	3.5 dB
column loss ($L_{C}$)	6.0 dB
antenna gain	3 dBi

**Table 3:** Results for the scenarios with D=21m.

Scen.	Active APs	Power [W]	Gain [%]	TN/AP	Airtime [%]
R	5.7	78.9	89.5	47.6	76.8
A1	3.1	40.8	86.4	35.3	62.1
A2	10.2	144.5	90.4	52.9	81.3
B1	5.2	72.5	90.3	26.1	47.9
B2	6.2	85.5	88.6	65.7	87.9
C1	5.7	78.8	89.5	47.6	74.8
C2	5.7	78.7	89.5	47.6	76.0
D1	5.6	78.0	89.6	48.0	57.0
D2	5.8	81.2	89.2	46.5	86.2

**Table 4:** Results for the scenarios with D=42m.

Scen.	Active APs	Power [W]	Gain [%]	TN/AP	Airtime [%]
R	21.1	287.6	61.7	12.8	30.2
A1	9.5	127.6	57.5	11.3	29.5
A2	39.4	541.9	63.9	13.7	32.7
B1	19.0	258.8	65.5	7.1	21.0
B2	22.3	305.3	59.3	18.1	39.8
C1	21.1	289.2	61.4	12.8	27.1
C2	21.1	287.0	61.7	12.8	29.3
D1	21.1	287.4	61.7	12.8	22.0
D2	21.2	288.9	61.5	12.8	36.8

**Table 5:** Solving CPU times (in seconds).

Scenario	D=21m	D=42m
R	4.9	1.8
A1	0.1	0.2
A2	269	42.9
B1	0.9	0.8
B2	129	18.7
C1	3.6	1.6
C2	5.2	1.9
D1	3.4	4.3
D2	23.6	138

**Table 6:** Quantised rates (in Mbps) for the reduced model energy (RME) model.

Ring	Power Level				
	k=1	k=2	k=3	k=4	k=5
r=1	54	54	54	54	52.8
r=2	33.1	27.8	22.5	17.3	12
r=3	12	6.7	1.4	0	0

**Table 7:** Comparison between BDA and RME.

Scenario	BDA	RME, $d_{3}$ = 40 m				RME, $d_{3}$ = 24 m		
	Time	Time	Power	Loss [%]	U.I. [%]	Time	Power	Loss [%]
R@21m	4.9	2320	88.0	11.6	70	63.1	182.8	133.5
A1@21m	0.1	2.5	40.8	0.2	25	1.8	83.9	105.6
A2@21m	269	3240	172	18.7	45	2839	342.4	139.5
B1@21m	0.9	5.4	72.3	−0.3	85	1.2	166.0	123.9
B2@21m	129	3240	106	23.4	15	916	187.9	122.7
C1@21m	3.6	2156	88.2	11.8	65	1.0	186.8	138.1
C2@21m	5.2	2663	88.1	11.9	85	71.9	182.7	133.4
D1@21m	3.4	46.5	77.8	−0.2	85	7.0	181.5	132.7
D2@21m	23.6	3241	99.9	23.0	80	199	184.5	132.1
R@42m	1.8	0.7	284.1	−1.2	90	–	–	–
